# How municipalities support energy cooperatives: survey results from Germany and Switzerland

**DOI:** 10.1186/s13705-020-00248-3

**Published:** 2020-03-18

**Authors:** Thomas Meister, Benjamin Schmid, Irmi Seidl, Britta Klagge

**Affiliations:** 1 Department of Geography, University of Bonn, Meckenheimer Allee 166, 53115 Bonn, Germany; 2 Swiss Federal Research Institute WSL, Zürcherstrasse 111, CH-8903 Birmensdorf, Switzerland

**Keywords:** Energy cooperatives, Municipal support, Community energy, National energy policy, Limiting factors, Survey results, Germany, Switzerland

## Abstract

**Background:**

Energy cooperatives are a prominent and common form of community energy. Community energy has the potential to increase actor diversity and local acceptance of renewable energies and has therefore been highlighted to be conducive to energy transitions. While research has recognized the importance of both the national and the local governance levels for community energy, it remains unclear how these two levels are related. Against this backdrop, this paper investigates how municipalities support energy cooperatives at the local level and how this support is related to national context conditions.

**Methods:**

The study takes a quantitative approach using own survey data from Germany and Switzerland. Based on a typology of municipal support, we compare limiting factors and municipal support for energy cooperatives between the two countries as well as between energy cooperatives with and without municipal membership. By means of this two-tiered comparison, we analyze how municipal support is related to national contexts, specifically regarding national energy policies, and to municipal involvement in the cooperatives.

**Results:**

Our analysis shows that municipal support can benefit energy cooperatives as it addresses some of the major limiting factors for energy cooperatives in Germany and Switzerland. However, our data suggest that municipalities only specifically address cooperatives’ limitations with support measures if they are a member in the cooperative. This indicates that organizational involvement of a municipality in energy cooperatives leads to a more targeted support compared to non-members and thus is beneficial to meet the specific cooperatives’ challenges due to national energy policy.

**Conclusions:**

Cooperatives can benefit from municipal support, especially if the municipality is a cooperative member. Municipal support is likely to become even more important for energy cooperatives in the future, due to reduced national support for renewable energies in Germany and Switzerland. On their part, municipalities can benefit from collaborating with energy cooperatives, as they gain an additional instrument to implement municipal energy policy. Hence, supporting and seeking membership in energy cooperatives appear to be adequate strategies for municipalities to foster a decentralized energy transition.

## Background

### Introduction

In the last two decades, “community energy” has emerged as a new phenomenon in various countries [1, 2] and has supported the development of renewable energies [3–5]. Community energy refers to locally or regionally embedded energy organizations with broad participation of citizens [2, 6–9], and its scope and form vary considerably between countries. Energy cooperatives are a prominent and common form of community energy in both Germany and Switzerland [10–12] and are the subject of our cross-country comparison.

In several studies, researchers tried to explain the diverging developments of community energy by focusing on factors at the national level [13–17]. They have shown that the development of community energy has been substantially shaped by the national regulatory frameworks and depends particularly on whether and how feed-in tariffs for renewable energies have been implemented [7, 13]. Other authors have emphasized the need for local acceptance and support for the development of renewable energy projects and thus the importance of local actors for community energy [18–20]. Municipalities are such actors. They can operate as collaboration partners, as shareholders in community energy organizations [21, 22], as network actors [23], as investors, or as buyers of the produced energy [24, 25]. With these different roles, municipalities are key actors for energy cooperatives.

While research has recognized the importance of both the national and the local governance level for community energy, it remains unclear how these two levels are related. This reflects a research gap in the discourse on multi-level climate governance: interactions between governance levels have been identified as relevant for policy effectiveness but still little is known about interactive effects of the levels [26, 27]. Against this backdrop, this paper focuses on support by municipalities for energy cooperatives at the local level and how such support is related to national context conditions.

We compare limiting factors and municipal support for energy cooperatives between Germany and Switzerland as well as between energy cooperatives with and without municipal membership within both countries. Based on such two-tiered comparisons, we analyze how municipal support is related to the national contexts, specifically regarding national energy policies, and to municipal involvement in the cooperatives. We show that municipal support complements national support policies, especially if municipalities are cooperative members.

We selected Germany and Switzerland for a cross-country comparison as both exhibit a widespread occurrence of community energy, which includes energy cooperatives. Both countries have federal and strongly decentralized political systems wherein municipalities—based on the principle of subsidiarity—have extensive responsibilities, some financial autonomy, and are expected to contribute to national and state energy policies. We can thus apply a comparative approach to examine municipal support for energy cooperatives in conjunction with national energy policies. Our focus is on energy cooperatives active in electricity generation since it is the most developed activity in the renewable energy sector of the two countries^1^ ([28, 29] for Germany, [30] for Switzerland).

In the following section, we review literature on energy cooperatives, their national contexts, and the roles of municipalities to illustrate the existing research gap and to refine the research questions. In the “Methods” section, we explain our research design, which is based on two comprehensive surveys of energy cooperatives in Germany and Switzerland, and introduce a typology of municipal support. We then compare the national contexts for energy cooperatives in Germany and Switzerland, providing the basis for the subsequent interpretation of our survey results. In the last two sections, we discuss the empirical results, and we conclude our study with a summary and policy recommendations as well as avenues for future research.

### Literature review: energy cooperatives, their national contexts, and the roles of municipalities

In the following, we introduce the cooperative model, its relevance for energy transitions, and how the development of such energy cooperatives is influenced by the national as well as the local level, especially by municipalities. The specific conditions for energy cooperatives in Germany and Switzerland are part of the empirical section, where they will be analyzed and compared with a focus on municipal support (cp. the “Analysis of the institutional contexts for energy cooperatives in Germany and Switzerland” section).

#### Energy cooperatives

Based on the fundamental principles of (collective) self-reliance and self-help, the first “modern” cooperatives were founded over 150 years ago as voluntary associations of people with the goal to pursue common economic, social, and cultural needs [31–33]. Cooperatives exist in various economic sectors, including the production of renewable energy [14].

Energy cooperatives are a common form of community energy, yet their numbers vary considerably between different countries: In Europe, by far the most energy cooperatives exist in Germany, Denmark, Austria, and Great Britain [1, 34], followed by Switzerland [35]. However, as cooperatives are not a specific legal form in some of these countries, numbers are only partially comparable and serve merely as a rough orientation.

Although energy cooperatives only own small shares of the nationally installed renewable energy capacity [8, 34, 35], they are widely considered as important actors in the energy transition due to strong citizen participation, to their democratic form of organization (one share one vote), and to their frequent pioneer role (e.g., electrification of rural areas, fostering transition to renewable energies). Hence, they are being perceived as representing public concerns [36–38]. More generally, their core principles are often associated with “openness, transparency and accountability” [39]. This may contribute to a high degree of social acceptance and perceived legitimacy of renewable energy projects [40], which are necessary for a successful transition towards a local sustainable energy system [15, 17, 38, 41, 42].

#### National context conditions for renewable energy and energy cooperatives

National support policies and the regulation of the electricity market are essential for the development of renewable energies [42, 43] and thus for energy cooperatives. Feed-in tariffs were found to be especially beneficial for the development of renewable energy, as they provide commercial feasibility of renewable energy projects and (long-term) planning reliability for project developers and investors. In contrast to other support policies such as tax credits or renewable portfolio standards, feed-in tariffs are easy to manage and independent from the scale of operations. They are thus conducive to the emergence and development of small energy producers such as energy cooperatives [6, 7, 15, 16, 44]. However, Dóci and Gotchev [13] as well as Nolden [7] show that the effectiveness of feed-in tariffs strongly depends on their specific design (e.g., regarding price setting) as well as the broader energy and planning policies (e.g., existence of soft incentives such as energy labels).

Also, national energy policies such as the regulation of the electricity market matter for the development of community energy: Low barriers to grid connection [16] as well as the opening of electricity markets for (small) companies and the promotion of competition (e.g., by liberalization and unbundling) [15] are mentioned as enabling factors for community energy. However, Kelsey and Meckling [45] found support and electricity market policy to be less important and argue that other factors such as resource endowments, relative technology prices, and the market effects of technological disruption determine which type of actors dominate the energy transition in a country.

Finally, a strong tradition of cooperative enterprises within a country—and therefore familiarity with the cooperative model—is an important if not necessary condition for the substantial development of energy cooperatives [6, 14, 16, 20]. Established cooperative institutions such as cooperative banks can also advance energy cooperatives. They share a common value framework, are regionally focused in their activities [46], and are often important creditors for energy cooperatives [46–48].

#### The role of municipalities in supporting energy cooperatives

Literature on multi-level climate and energy governance stresses the interplay between different levels of governance and specifically the important role of local governments [49–51]. Hence, several authors see municipalities (local governments) as important actors for local energy producers [24, 52] such as energy cooperatives, whose activities and members are usually locally embedded [53, 54]. Municipalities may function as network actors [23] and operate as collaboration partners or shareholders [21, 22] and as investors or buyers of the produced energy [24, 25]. Furthermore, municipalities can have a beneficial impact on creating trust and adding legitimacy to energy initiatives planned by (local) actors, such as energy cooperatives [18, 41, 55].

Overall, only a few studies have analyzed the role of municipalities for energy cooperatives and, more generally, for community energy. Mey et al. [25] conducted a survey of local governments in Australia and found that local governments support community renewable energy in various roles: as facilitators (e.g., by purchasing energy), as innovators and participants, as catalysts and supporters, and as networkers and advocates. Furthermore, they identified the local governments’ motives for support of, and cooperation with, community energy: mobilization of an active citizenry, enhancing their reputation, and meeting policy targets. Herbes et al. [44] showed that local policy makers may engage in partnerships with energy cooperatives to advance their energy policy goals but that municipal energy utilities, on the other hand, can consider energy cooperatives as competitors. Hoppe et al. [18] compared two successful local energy initiatives, one in Germany and one in the Netherlands, and highlighted the roles of municipalities as initiators and network actors as well as mediators between local stakeholders. Moreover, they found differences regarding the degree of organizational involvement of municipalities in the decision-making process of the energy initiatives. This is also reflected in Edelenbos et al. [56], who focus on “community self-organization” and show that its evolution strongly depends on the interaction with local governments.

### Research questions

The literature review shows that both the national and the local governance level contribute to the development of community energy organizations in general and energy cooperatives in particular. However, it remains unclear how these two levels are related regarding the support of energy cooperatives. To address this research gap, we focus in this paper on municipal support for energy cooperatives and analyze it in conjunction with national energy policies in Germany and Switzerland. We also investigate the factors which the energy cooperatives saw as limiting for their development thus far and in the future in order to put the analysis of municipal support into perspective.

Overall, we ask: *How do municipal support measures and limiting factors for energy cooperatives depend on national context conditions and on the membership of municipalities in energy cooperatives*? This research question is answered in three steps with corresponding sub-questions:

The first step concerns the institutional contexts for energy cooperatives in Germany and Switzerland and draws on a literature analysis. Given institutional factors are relevant as the literature review of above shows, we ask the following: (i) What characterizes the German and Swiss energy policy in terms of electricity market regulation and support policies for renewables? (ii) What are the responsibilities and competences of municipalities in the German and Swiss federalist systems when it comes to energy policy? (iii) What is the legal status of cooperatives and is there a tradition of energy cooperatives in the two countries?

The second step is based on the analysis of our survey data and concerns the specific support provided by the municipalities and the perceived limiting factors by energy cooperatives. We ask the following: (i) How do municipalities support energy cooperatives and how does municipal support differ between Germany and Switzerland and between cooperatives with and without municipal membership? (ii) What do energy cooperatives in Germany and Switzerland perceive as major limiting factors and do the perceived limiting factors differ between Germany and Switzerland and between cooperatives with and without municipal membership?

In the third step, our survey results regarding municipal support and perceived limitations are interpreted and discussed in light of the different institutional contexts in the two countries. We ask the following: (i) How is municipal support related to national context conditions? (ii) Are limiting factors perceived by the energy cooperatives related to national context conditions or differences in municipal support?

This approach enables us to connect the national with the local level and thus to address the identified research gap. In the next section, we specify the methodical approach.

## Methods

This study takes a quantitative approach using own survey data from Germany and Switzerland. In what follows, we provide information on how we gained and analyzed the data, we present basic data, and we present a typology that differentiates forms of municipal support.

### Survey design, data analysis, and quality

Our comprehensive surveys in both Germany (DE) and Switzerland (CH) were built on previous surveys and on experts’ workshops and interviews. Based on the cooperative register in Germany, on the Swiss trade registry, and on internet research in both countries, we identified 828 active energy cooperatives in Germany and 289 in Switzerland. These cooperatives were contacted by mail in the second half of 2016 with a questionnaire and a link in case an online format of the survey was preferred. The questionnaire addressed members of the executive or supervisory board of the cooperatives as these are directly involved in major business and organizational matters. After two reminders per mail, 213 German and 136 Swiss cooperatives had participated in our survey up to March 2017, resulting in response rates of 25% in Germany and 47% in Switzerland. The response rate in Germany is in line with similar recent surveys [21, 37, 47, 57]; in Switzerland, it was the first survey of energy cooperatives.

To assess the representativeness of the overall data, we compared the foundation year of the answering cooperatives with those in other survey data sets. The age structure of the surveyed German cooperatives is to a large degree similar to that in other surveys [21, 47] and to a comprehensive data collection based on the cooperative register [10]. The Swiss survey data reflects the general pattern of foundation years published in the Swiss trade register, although it does not match as well as in the German case.

In order to ensure comparability between the German and Swiss energy cooperatives, we only used a sub-sample of all the surveyed cooperatives, applying two filter criteria. The first filter concerned the time of the cooperatives’ formation: 98% (208 out of 213) of the responding cooperatives in Germany were founded in or after 2006, which is closely related to major legislative changes in German cooperative law in 2006 (see also below). In contrast to Germany, many energy cooperatives in Switzerland were founded before 2006. Nonetheless, we only included cooperatives which were founded in or after 2006 to ensure the comparability of data and results as the institutional settings and the technological development changed significantly between the beginning of the 1990s and 2006 in both countries. The second filter concerned the kind of activity: Renewable electricity generation was the main activity of the cooperatives in both our surveys. Among the surveyed cooperatives founded after 2006, 77% (160 out of 208) of German cooperatives and 77% (50 out of 65) of Swiss cooperatives were active in renewable electricity generation (operator of own facilities) in 2016 (or plan to do so in the immediate future). Considerably fewer energy cooperatives in both countries were (also) engaged in other activities, such as heat generation (21% in DE; 28% in CH).

The selected data of our sub-sample were analyzed in two steps: Firstly, we compared municipal support and limiting factors for energy cooperatives between Germany and Switzerland. Secondly, we compared municipal support and limiting factors between cooperatives with and without municipal membership within each country (Table 1). We used the software SPSS to evaluate the data. Since we only examined dichotomous variables, we conducted chi-square tests^2^ to test for the significance of differences between Germany and Switzerland and cooperatives with and without municipal membership respectively. We used Cramer’s *V* to classify the extent of group differences in .10, .30, and .50 corresponding to small, medium, and large respectively [59]. For the results, see Supplementary Table A.1, Additional file 1.

**Table 1:** Comparisons in analysis

	Comparison between:	
	Germany and Switzerland	Cooperatives with and without municipal membership within Germany and Switzerland
Comparison of:	• Perceived limiting factors (thus far and in the future) • Municipal support	• Perceived limiting factors (thus far and in the future) • Municipal support

Our statistical analyses provide us with relative frequencies and statements on the statistical significance of differences. We interpret these results in light of a comparison of the (national) institutional contexts for energy cooperatives in Germany and Switzerland. Given the factors identified in the “Literature review: energy cooperatives, their national contexts, and the roles of municipalities” section, these contexts include the German and Swiss energy policy, the responsibilities and competences of municipalities in the federalist systems, cooperative law, and the tradition of (energy) cooperatives. For this comparison of institutional contexts, we rely on existing literature and governmental documents.

### Selected survey data on energy cooperatives in Germany and Switzerland

In the following, we provide some basic characteristics of the German and Swiss energy cooperatives in our sub-sample to help the interpretation of the results and compare our data to other surveys and publications. Photovoltaics (PV) was by far the most commonly used technology in both countries, with a slightly lower share in Germany (86%; 136 out of 158) than in Switzerland (93%; 41 out of 44). Other renewable electricity technologies were scarcely used. Only 19 German cooperatives produced electricity from wind power (median of installed wind capacity, 4800 kW), and 13 out of these 19 also used PV. In the Swiss data set, it was only one, and it also used PV. Due to the predominance of PV in cooperative electricity generation, we interpret our results primarily against the background of this technology.

Our survey data show considerable differences between energy cooperatives in Germany and Switzerland (Table 2): German energy cooperatives were much larger in terms of the number of members, installed capacities (PV), and balance sheet total (indicating the size of the cooperative). Despite this difference, the cooperatives in both countries had in common that their aggregated share of total installed PV capacity amounted to about 1–1.5% in 2016 (own estimate^3^).

**Table 2:** Comparison of cooperatives in Germany and Switzerland. Source: Own surveys

Indicators		Country		Municipal membership			
				Germany		Switzerland	
		Germany	Switzerland	Yes	No	Yes	No
Number of members (private and institutional)	Median	147	47	147	152	57	38
	Mean	252	76	266	247	95	58
	*N*	*156*	*49*	*87*	*58*	*24*	*25*
Installed PV capacity (kWp)	Median	254	109	242	262	132	55
	Mean	1035	208	1245	659	279	139
	*N*	*130*	*41*	*71*	*47*	*20*	*21*
Balance sheet total 2015 (in 1000€)*	Median	841	200	705	948	299	100
	Mean	2419	894	2361	1934	949	841
	*N*	*143*	*35*	*82*	*51*	*17*	*18*

*Exchange rate (31.12.2015): 1 CHF = 0.9196 Euro

Finally, member groups differ (Table 3). Whereas municipalities are a common member group in both countries, other key member groups, like cooperative and other banks as well as utilities, are common member groups only among German energy cooperatives.

**Table 3:** Comparison of selected member groups in Germany and Switzerland. Source: Own surveys. Example for interpretation: 60% of German cooperatives (89 out of 148) had at least one municipality as a member

Member groups		Country		Municipal membership			
				Germany		Switzerland	
		Germany	Switzerland	Yes	No	Yes	No
Municipality	Share (*x* out of *N*)	60% 89/148	50% 25/50	–	–	–	–
Cooperative banks	Share (*x* out of *N*)	53% 79/148	8% 4/50	73% 65/89	24% 14/59	16% 4/25	0% 0/25
1.13. Other banks	Share (*x* out of *N*)	26% 39/148	2% 1/50	37% 33/89	10% 6/59	4% 1/25	0% 0/25
1.20. Energy utility	Share (*x* out of *N*)	34% 50/148	12% 6/50	48% 43/89	12% 7/59	24% 6/25	0% 0/25

To assess the representativity of our sub-sample data, we compared them with the results of other surveys. However, we only had comparison data for the German cooperatives as our survey was the first of its kind in Switzerland.

Our data on the German cooperatives is similar to data from other surveys on energy cooperatives in Germany [10, 37, 41, 47, 57, 61, 62] in terms of the average number of members [47], the installed PV capacity [61], and the member structure. Also, the importance of municipalities is clearly reflected in other studies: In a study by Volz [57], 63% of the surveyed energy cooperatives mentioned municipalities as members, and in a study by Debor [41], 41% of the surveyed energy cooperatives even referred to municipalities (communities) as dominant collaborative partner (founding partner or member of a cooperative’s steering board). But there are also differences: in the survey by Volz, a considerably higher share of energy cooperatives mentioned cooperative banks as members (73%) and a lower share mentioned other local banks as a member group (12%). As Volz’s study was conducted as early as 2012, an explanation for this discrepancy could be that at this time cooperative banks took on a pioneer role in promoting energy cooperatives in Germany (cp. Hall et al. [46] and the “Energy cooperatives” section) and other (local) banks followed their lead later. Debor [41] also highlighted the importance of “banks, particularly cooperative ones” (p. 149) as dominant collaborative partners (33%). Moreover, both studies showed that energy-related companies (such as municipal utilities) are another important member group. In summary, this brief comparison shows that our sub-sample data are very similar to other surveys and publications. We therefore expect our data also to be representative with respect to municipal support, which has not yet been quantitatively analyzed in other surveys.

### Typology of municipal support for energy cooperatives

**Fig. 1:**
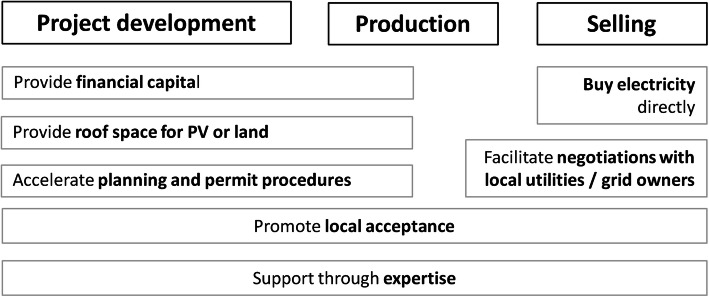
Typology of municipal support for energy cooperatives. Source: Own design

Municipal support for energy cooperatives can address different topics and activities. Hence, we developed a typology of municipal support based on existing literature and expert interviews (Fig. 1). This typology guides the empirical analysis. It distinguishes forms of municipal support along the development stages of renewable energy projects and related activities which may be subject to municipal support. These development stages are as follows: (1) *project development*, (2) *production*, and (3) *selling* of the produced energy. As municipal support can help to overcome various (potential) obstacles [44], the typology not only reflects forms of municipal support but also the limiting factors that may arise in these development stages.

The *project development* stage encompasses planning, design, installation, and implementation of the facility. The investor, project developer, or future power producer must secure capital, suitable land/roof space, and permits and must decide on technologies, commercialization, and organizational structures. Also, there may be opposition against renewable energy projects which must be addressed [55, 63, 64]. In this stage, municipalities may support energy cooperatives in the following ways (Fig. 1): They can provide capital, for instance as member of a cooperative (thus contributing equity) or through loans or guarantees, and they may make available land or roof space on public buildings. As a legal authority, they may accelerate planning and permit procedures. By being a cooperative member, they may advance acceptance, not least because they are often involved in various networks and may have trustful relationships with central stakeholders. Hence, municipalities may have a mediating and legitimizing role in cases of conflict and opposition and thus may promote acceptance of projects and their operations, including production and selling.

The *production stage*, i.e., the generation of electricity, is rather simple for PV (cooperatives’ most used technology). Municipal support options are limited and include support for local acceptance if there are production-related conflicts.

The activities in the *selling stage* depend on the market structure and regulation. A power producer can sell electricity to utilities, to end-consumers, or to electricity exchanges. In this stage, municipalities may support energy cooperatives as follows: they may buy electricity or facilitate negotiations with the local utility and/or grid owner, by using their legal authority.

Finally, in all three stages, municipalities may provide expertise, including experience and contacts.

Our typology shows that municipalities can support energy cooperatives in all development stages of renewable energy projects. However, the capabilities of municipalities to implement support structures strongly depend on the specific national context, including the electricity market and federal regulations as well as the acceptance and legitimacy of the cooperative model. These aspects are analyzed in the following section for Germany and Switzerland.

## Results and discussion

### Analysis of the institutional contexts for energy cooperatives in Germany and Switzerland

Electricity market regulations and national support policies for renewable energies differ considerably between Germany and Switzerland. On the other hand, the role of municipalities in the federal systems of the two countries and the cooperative law and tradition of (energy) cooperatives are rather similar (for an overview, see Table 4). In this paper, we refer to the national context conditions up to 2016, the year of our survey.^4^

**Table 4:** Institutional contexts for energy cooperatives in Germany and Switzerland

	Germany	Switzerland
RE support policies	• Feed-in tariff: EEG 2000, 2004, 2009, 2012, 2014, 2016/2017	• MKF (2005) • Capped feed-in tariff: KEV (2009) • One-off inv. grants (2014)
Electricity market regulation	• High level of liberalization: Competition among electricity retailers, free choice of electricity suppliers for customers, unbundled generation, regulated transmission grids operated by four regional operators	• Low level of liberalization: Limited competition among electricity retailers and limited choice of electricity suppliers for customers (area monopolies for small consumers (< 100 MWh))
Municipalities in the federal system	• Lowest administrative level • High degree of local autonomy with limited financial autonomy • Own municipal energy policies	• Lowest administrative level • High degree of local autonomy with extensive financial autonomy • Own municipal energy policies
Cooperative law and tradition	• Cooperative law (2006) • Pronounced cooperative tradition (housing, retail trade, energy)	• Swiss Code of Obligations • Pronounced cooperative tradition (housing, retail trade, energy)

#### Electricity markets and national support policies for renewable energies

In Germany, the rapid development of renewable energies in the last two decades has been driven by three changes in national energy policy: (i) the liberalization of the electricity market following EU regulations in the 1990s, (ii) the implementation of the Renewable Energy Act (EEG, Erneuerbare Energien Gesetz) in 2000, and (iii) the decisions to phase-out nuclear electricity production in 2000 and 2011. Liberalization included unbundling, abolition of area monopolies, and opening of grid access. The EEG introduced feed-in tariffs (FITs) without a cap for renewable electricity generation (costs are passed on to the customers). The decisions to phase-out nuclear power were based on long socio-political discussions. All these changes brought the share of renewable energy production to 29% of the gross electricity generation in 2016 [60]. The production has been rather decentralized and characterized by a large variety of different, often small-scale players [65]. Yet, large projects have increased in numbers recently. One reason is the amendments of the EEG in 2014 and in 2016/17: FITs were gradually phased out and the mandatory direct marketing for newly approved renewable energy facilities (> 100 kW) was introduced. Moreover, tender procedures for renewable energy facilities (> 750 kW) were implemented. Especially small-scale actors, such as energy cooperatives, are therefore challenged by more complex regulations, a stronger market orientation, and increased competition [41, 44].

Compared to Germany, Switzerland had no fundamental changes in its energy policy by 2016. Electricity market liberalization concerned only large consumers (> 100 MWh/year), whereas smaller consumers still are bound to the (local) power suppliers [66] and thus to territorial monopolies. As a result, producers which are not (local) electricity suppliers themselves need to sell their electricity to the electricity supplier and are prevented from selling it to small consumers. Political instruments to foster renewables were modest: The instrument “financing of additional costs” (MKF) of 2005 hardly affected the renewable energy production [67], and a new feed-in tariff (KEV^5^) of 2009 featured a capped total available budget, which led to a long waiting list and uncertainty regarding KEV funding. In 2014, “one-off investment grants” were implemented as an alternative to KEV funding. They cover up to 30% of investments costs of PV installations below 30 kW. Due to this limited financial support and confined market, energy cooperatives had always been dependent on their own distribution channels within the territorial monopolies [35].

In 2011, Switzerland decided to phase-out nuclear power, which also underlined its will to foster renewable energies. In 2016, the overall share of renewables was at 62.3% of electricity generation (57% was hydropower, and 2.3% solar power) [30].

#### Municipalities in the federal systems of Germany and Switzerland

Although Germany and Switzerland differ in their types of federalism [68, 69], in both countries, the sub-national level holds considerable powers. This concerns both the state level (*Länder* in Germany and *cantons* in Switzerland) and municipalities. Municipalities in both countries share a high degree of local autonomy, including certain financial autonomy [70] and legal authority in planning and approving of renewable energy facilities [71]. Also, they often own local energy utilities. However, the financial autonomy of German municipalities is comparatively more limited, and their activities are more specifically regulated, with economic activities only being permitted if they serve the public interest and if they lie within the (financial) capacities of the municipalities. Some of the federal states’ municipal codes require an “appropriate” influence for participation in private enterprises [24, 72]. Overall, municipalities may—within narrow legal bounds—engage economically in the energy sector since electricity production and distribution are regarded as a matter of public interest [24].

In Germany, the federal and state levels are responsible for policies in energy and spatial planning. This includes, for example, the electricity market design, renewable energy targets, and general rules of planning. Municipalities develop and implement their own energy policies and decide on land use planning and building permits within the federal hierarchy. As renewable energy facilities, apart from most roof-PV systems, usually require permits, municipal approval is indispensable [24].

In Switzerland, energy policy at the national level was only constitutionalized in the 1990s and typically consists of general political targets, framework laws, and programs [73]. Historically, cantons and municipalities have been key actors in energy provision and policy and still play a substantial role in implementing federal energy targets. The same applies to spatial planning and issuing building permits. Similar to Germany, municipalities have substantial autonomy in deciding on their own energy policies—within the federal hierarchy, their financial capacities and often with support from the federal and cantonal level.

In summary, municipalities in both countries occupy similar key positions when it comes to supporting and developing renewable energy projects and implementing energy policies, even though Swiss municipalities enjoy larger fiscal autonomy and financial self-reliance [74].

#### Cooperative law and tradition of (energy) cooperatives

**Fig. 2:**
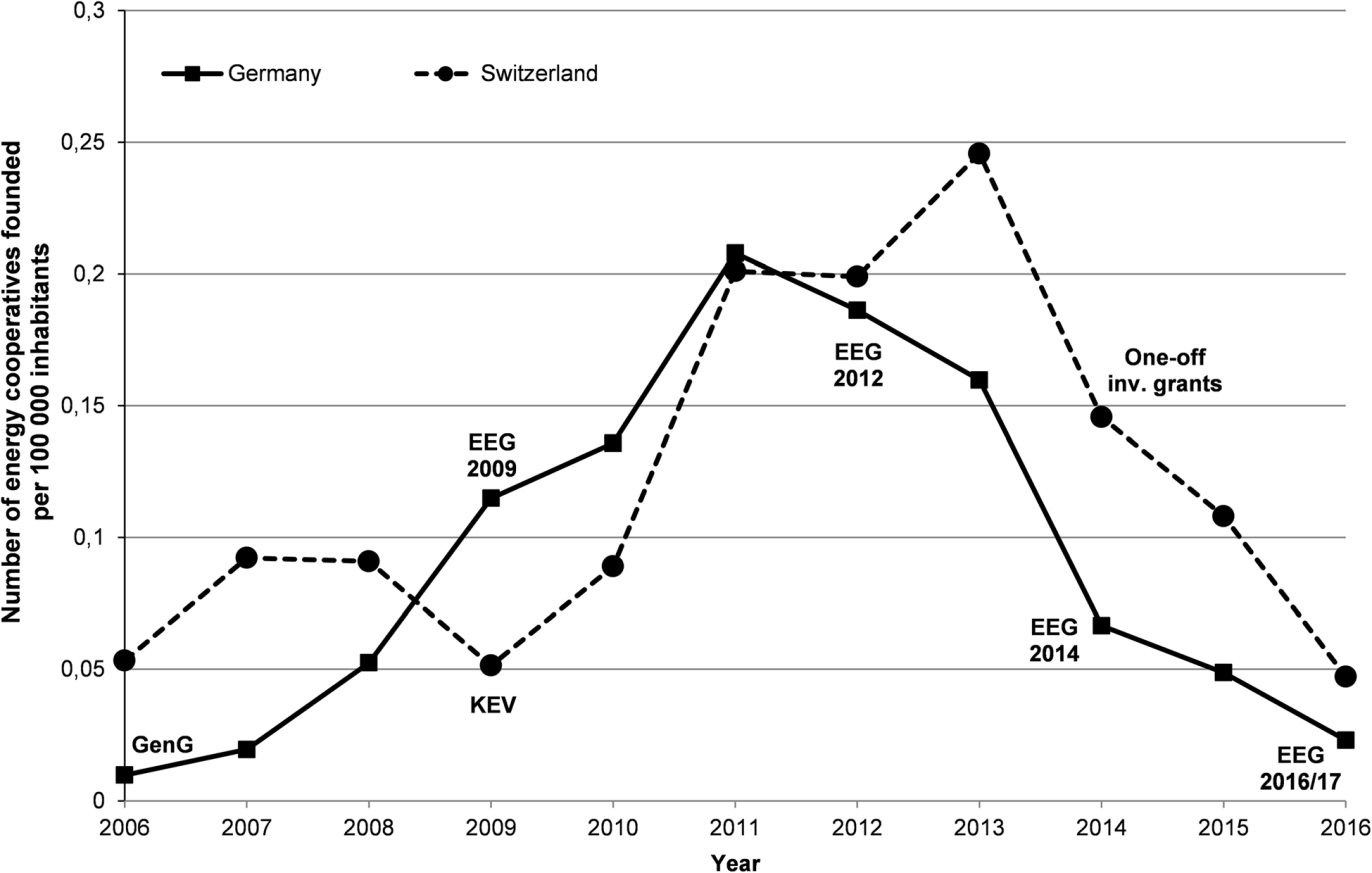
Foundations of energy cooperatives per 100,000 inhabitants and year (adapted from Schmid et al. [81])

In Germany and Switzerland, particular laws or provisions stipulate the conditions under which cooperatives operate. In both countries, cooperatives are a well-known and trusted organizational form of enterprise [12], and there is a large number of cooperatives active in sectors such as housing, retail trade, and energy [31, 75–77]. The involvement of cooperatives in the energy sector goes back to the rural electrification at the end of the nineteenth/beginning of the twentieth century [57, 78, 79]. Of that time, in Switzerland more than 100 cooperatives are still active as distribution grid operators [11]. However, most energy cooperatives have been forced out of the market by public, private, and privatized utilities [11, 14, 80, 81], a process that was even stronger in Germany. Only recently have energy cooperatives experienced a renaissance in both countries. Figure 2 shows the number of newly founded renewable energy cooperatives per 100,000 inhabitants since 2006 and indicates relevant changes in the regulatory frameworks.

In Germany, the emergence and remarkable increase of renewable energy cooperatives (Fig. 2) was facilitated by the EEG and an amendment of the cooperative law in 2006, which simplified the rules and requirements to found (energy) cooperatives. Since then, the most important business model of energy cooperatives was renewable electricity generation based on the earlier established support policies for renewable energies, i.e., using FITs as stipulated by the EEG [37, 57]. Hence, the development of energy cooperatives has remained closely associated with EEG and its various amendments. More recently, the formation of new energy cooperatives has been curbed: by the changes regarding the support of PV (EEG 2012), by temporary uncertainties regarding capital investment regulations [21, 82], and, more recently, by the phasing-out of feed-in tariffs (EEG 2014 and EEG 2016/2017). Despite these changes, electricity generation based on FITs was and still is a widespread business model of energy cooperatives, with a strong focus on PV and, to a much lesser extent, on wind [54].

In Switzerland, renewable energy cooperatives first emerged in a large wave between 1990 and 2000 [35]. A second, larger wave of new energy cooperatives emerged between 2006 and 2012 (Fig. 2). The start of this wave runs parallel to the development of national renewable energy support schemes, in particular the KEV funding (FIT) (see above). After peaking in 2012, the number of new cooperatives has since decreased. A possible reason is the minimal chance of access to feed-in tariffs and the uncertainty about the political direction of support schemes. The vast majority of new cooperatives founded since 2006 generate electricity using mostly PV, with only a few relying on other technologies, or focusing on heat generation.

To sum up, this comparison shows that there is a similarly long tradition and a similar dynamic with respect to recent foundations of energy cooperatives in Switzerland and Germany.

### Survey results: municipal support and limiting factors for German and Swiss energy cooperatives

**Fig. 3:**
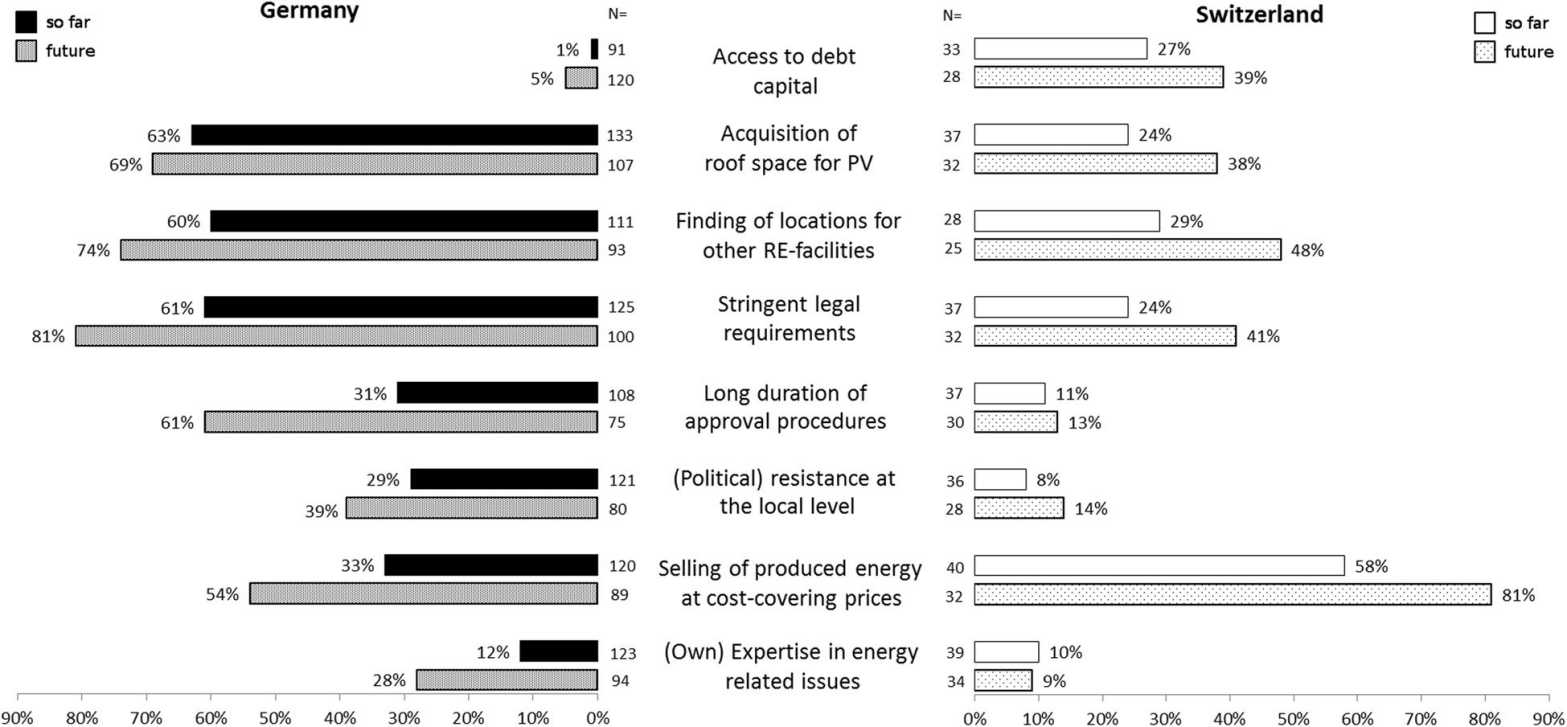
Major limiting factors (so far and in the future) perceived by energy cooperatives in Germany and Switzerland. Source: Own surveys

**Fig. 4:**
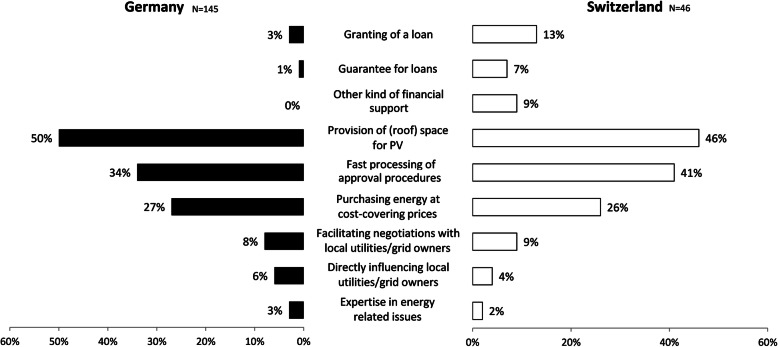
Overview of municipal support in Germany and Switzerland. Source: Own surveys

The presentation of the results is structured along the identified types of municipal support (Fig. 1) and the two-tiered comparison (Table 1). Figure 3 provides an overview of the limiting factors energy cooperatives face in both countries, and Fig. 4 presents an overview of the municipal support measures.

#### Financial support

Municipalities can provide financial support to cooperatives in the form of equity or other financial support such as loans or guarantees: In terms of equity, municipalities can become members and buy one or more shares. In Germany 60% and in Switzerland 50% of the renewable energy cooperatives had municipalities as members (Table 3). This made them the second most common member group after private individuals.

**Fig. 5:**
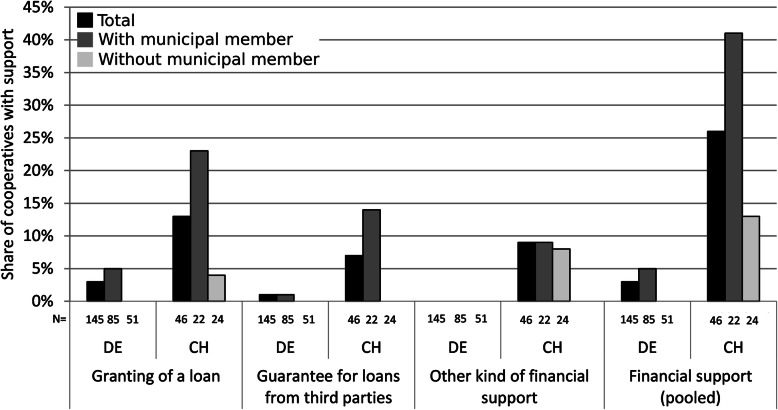
Municipal financial support for energy cooperatives with and without municipal membership in Germany and Switzerland. Source: Own surveys

In terms of other financial support, municipalities can—as members and non-members—financially support cooperatives by granting loans or providing guarantees. In Germany, both kinds of financial support by municipalities were uncommon (Fig. 5): Fewer than 3% of the cooperatives were granted loans by a municipality (all with municipal membership) and only one (< 1%) was given a guarantee by a municipality. No German cooperative mentioned any other kind of financial support (e.g., through an energy fund). Thus, financial support by municipalities in Germany was mostly limited to membership and thus providing equity.

The situation was quite different in Switzerland (Fig. 5). In Switzerland, financial support by municipalities (apart from membership) was significantly more frequent than in Germany and, within Switzerland, significantly more frequent in cooperatives with municipal membership (for *χ*^2^ tests, see Supplementary Table A.1, Additional file 1): 13% of the cooperatives (mostly with municipal membership) were granted loans by a municipality and 7% (all with municipal membership) were provided a guarantee by a municipality. Furthermore, 9% of the cooperatives were financially supported by municipalities through municipal energy funds or in other ways. In summary, municipalities in Switzerland financially supported cooperatives more often than in Germany (3% versus 26% of cooperatives), especially if a municipality was a member.

This difference between German and Swiss cooperatives reflects both the use of debt capital and the perceived difficulty of obtaining it (Table 5 and Fig. 3). Whereas in Germany 79% of the energy cooperatives used debt capital, the corresponding figure for Swiss cooperatives was 54% and thus significantly smaller (yet with similar mean debt ratios of 57% [DE] and 56% [CH], respectively). Accordingly, almost half of Swiss cooperatives assessed the acquisition of debt capital as very or rather difficult (44%) whereas for German cooperatives, it was the case for less than a quarter (23%). This is also reflected in the assessment of access to debt capital as a major limiting factor (Table 5 and Fig. 3).

**Table 5:** Financial characteristics of energy cooperatives in Germany and Switzerland. Source: Own surveys

Variables	Country					
	Germany			Switzerland		
	Total	Municipality		Total	Municipality	
		Member	No member		Member	No member
Use of debt capital	79% (124 out of 157)	81% (71 out of 88)	77% (44 out of 57)	54% (25 out of 46)	64% (14 out of 22)	46% (11 out of 24)
Difficulties to raise debt capital (very or rather difficult)	23% (30 out of 128)	24% (17 out of 72)	24% (11 out of 46)	44% (11 out of 25)	37% (6 out of 16)	56% (5 out of 9)
Loans from cooperative banks	51% (80 out of 156)	55% (48 out of 87)	46% (26 out of 57)	11% (5 out of 46)	23% (5 out of 22)	0% (0 out of 24)

Most likely, German banks more easily granted debt capital for renewable energy projects compared to Swiss banks, as the German EEG guaranteed a supportive financial framework, thus resulting in a low level of credit risk [48]. Moreover, a study by Hall et al. [46] showed that there is “a dense network of locally rooted banking institutions” (p. 12) in Germany which support enterprises like cooperatives. This is reflected in our data: banks, whether cooperative or otherwise, were more often members in German than in Swiss cooperatives (Table 3), and more than half of the German cooperatives had loans from cooperative banks (51%) compared to only 11% in Switzerland (Table 5).

#### Provision of land or roof space

**Fig. 6:**
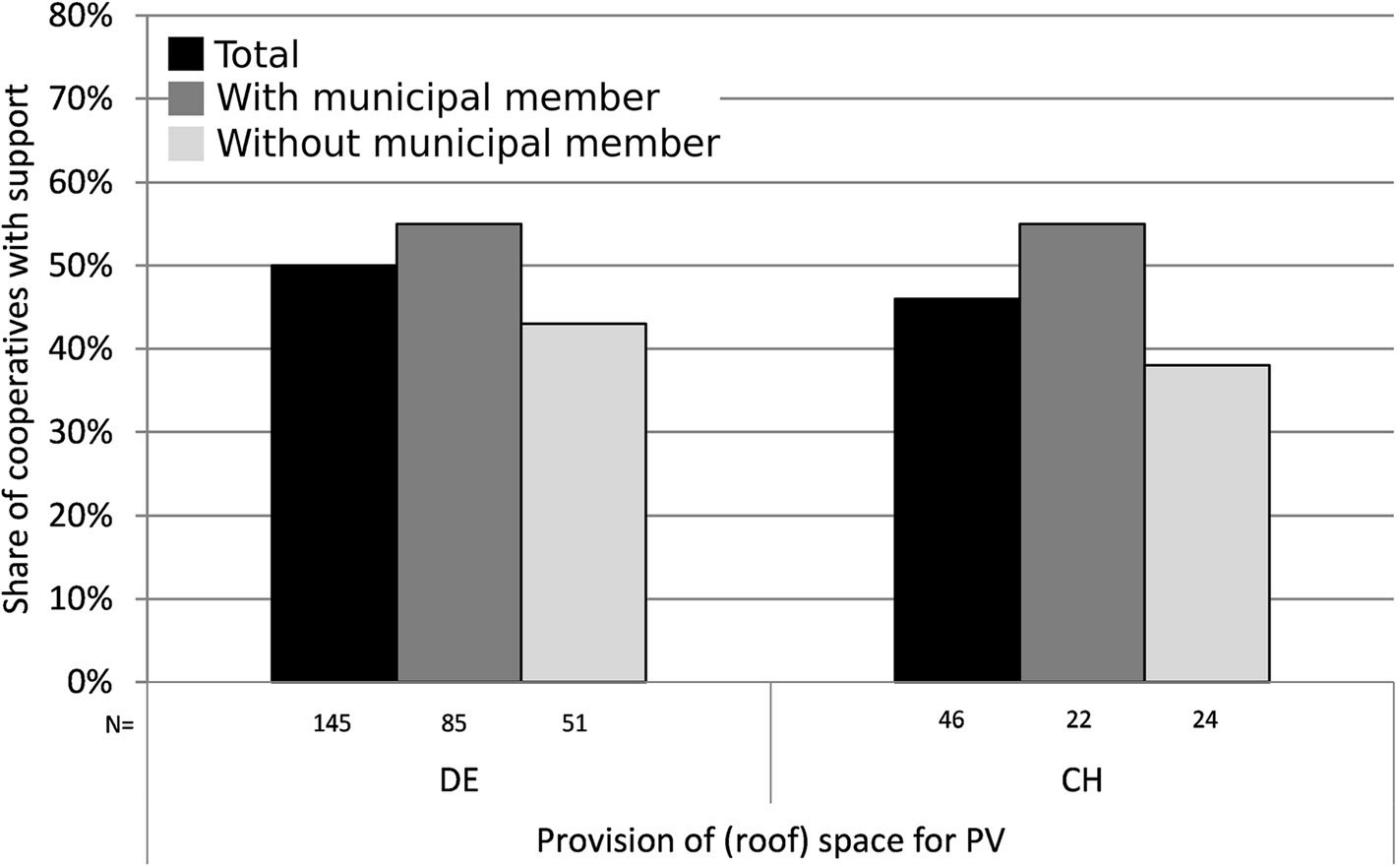
Municipal provision of roof space or land for energy cooperatives with and without municipal membership in Germany and Switzerland. Source: Own surveys

Renewable energy installations are land-intensive, which is why the acquisition of suitable land or roof space is often discussed as a limiting factor [83, 84]. For Germany, this was reflected in our data: A large majority of German cooperatives (63%) assessed the acquisition of roof space for PV as a major limiting factor. In contrast, Swiss cooperatives mentioned the acquisition of roof space significantly less often as a major limiting factor (Fig. 3). One reason for this difference may be related to the larger number and size of already developed renewable energy projects in Germany, which made it more difficult to find suitable areas and more prone to conflicts with competitors for such areas. Besides the spatial restrictions, opposition may be another obstacle for land or roof space acquisition (cp. the “Promotion of local acceptance” section). As municipalities often own large buildings suitable for PV, they are able to support cooperatives by providing (roof) space. In Germany, this kind of support was mentioned by 50% and in Switzerland by 46% of the surveyed cooperatives. In both countries, this support was slightly more frequent if a municipality was a member, albeit not significantly (Fig. 6).

#### Support in planning and permit procedures

Stringent legal requirements and a long duration of approval procedures due to objections of third parties may be major limiting factors for project development—especially for small enterprises like cooperatives. Our data show that this was widespread in Germany (Fig. 3). The majority of energy cooperatives mentioned stringent legal requirements (e.g., technical or environmental standards in project development) as a major limiting factor, especially for the future.^6^ Whereas a minority mentioned a long duration of approval procedures due to (legal) objections by third parties as a limiting factor thus far (31%), the majority (61%) of German cooperatives assessed this as a future obstacle. In contrast, most Swiss cooperatives did not assess these two factors to be major limiting factors. Only 24% of Swiss energy cooperatives mentioned stringent legal requirements as previous and 41% as future limiting factor, and the percentages are even lower for the delay in the approval procedure due to (legal) objections by third parties (Fig. 3).

**Fig. 7:**
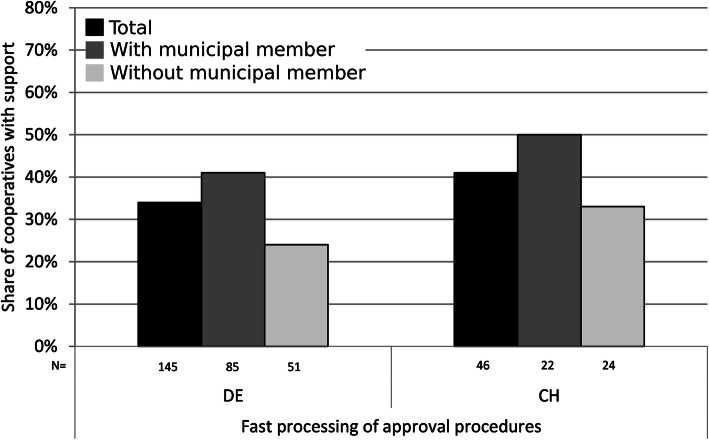
Municipal support in administrative procedures with and without municipal membership in Germany and Switzerland. Source: Own surveys

In both countries, municipalities are largely responsible for the planning and approval procedures of (large scale or greenfield) renewable energy facilities and can—within narrow legal bounds—support cooperatives through administrative procedures (cp. the “Municipalities in the Federal Systems of Germany and Switzerland” section). Although the Swiss cooperatives mentioned municipal support with respect to fast processing approval procedures slightly more often (41%) than German cooperatives (34%; Fig. 7), this difference was not significant (see Supplementary Table A.1, Additional file 1).

We expected that cooperatives with municipal membership benefit more often from such support and that they assess legal requirements and delayed approval procedures less often as major limiting factors. Our data supported this expectation but only in Germany: German cooperatives with municipal membership assessed stringent legal requirements and long approval procedures significantly less often as major limiting factors than cooperatives without municipal membership (see Supplementary Table A.1, Additional file 1). This finding is in line with the fact that cooperatives also mentioned municipal support through fast approval procedures more often if a municipality was a member (41% vs. 24%). Hence, the data indicate that the membership of a municipality in German energy cooperatives is beneficial for the approval procedures. This is not necessarily due to an active “fast-tracking” of the approval procedures but may be a result of expertise provided by municipal members to the cooperative (cp. the “Cooperation and expertise” section). Interestingly, we could not find the same result among the Swiss cases.

#### Promotion of local acceptance

The development of renewable energy projects is often met with resistance at the local level. This may concern the set-up of the facilities, aesthetical considerations, ensuing enlargement of the grid and other infrastructure installations, support by municipalities, (non)distribution of gains, etc. However, in Switzerland, this was rarely mentioned as a major limiting factor thus far (8%) or foreseeably in the future (14%).^7^ In contrast, German cooperatives mentioned resistance significantly more often as a major limiting factor thus far (29%) and even more so as a future obstacle (39%). These differences might be related to the higher number and (on average) larger size of already existing renewable energy facilities in Germany (cp. the “Provision of land or roof space” section).

In Switzerland, resistance was hardly rated as a major limiting factor, and we could not assess whether municipal membership makes any difference in this regard. In Germany, on the other hand, there was a significant negative association (see Supplementary Table A.1, Additional file 1) between the membership of a municipality and local resistance as a perceived major limiting factor: Fewer than 20% of the German cooperatives with municipal membership mentioned local resistance as a major limiting factor as opposed to nearly 42% without municipal membership. This difference was even more striking when it came to the assessment of local resistance as a major limiting factor in the future (29% vs. 56%). In summary, resistance was a much more important topic for the German than the Swiss energy cooperatives, especially for those without municipal membership.

#### Purchase and support for selling of electricity

The electricity markets and national support policy instruments for renewable energies differ considerably between Germany and Switzerland (cp. the “Electricity markets and national support policies for renewable energies” section). Likely due to the limited coverage of the Swiss feed-in tariff (KEV), the sale of electricity was significantly more often perceived as a major limiting factor thus far in Switzerland as compared with Germany (58% vs. 33%), where energy cooperatives had so far benefitted from the comprehensive FIT as provided by the EEG. However, the future assessment of sale possibilities was much more pessimistic in both countries. In Germany, 54% of the respondents (an additional 21%) mentioned the sale of energy at cost-covering prices as a major limiting factor in the future, anticipating the implications of the recent changes in the regulatory framework in Germany (even though existing facilities are still entitled to FITs). In Switzerland, the increase was even larger: 81% mentioned the sale of produced energy at cost-covering prices as a likely limiting factor in the future (an additional 23%).

**Fig. 8:**
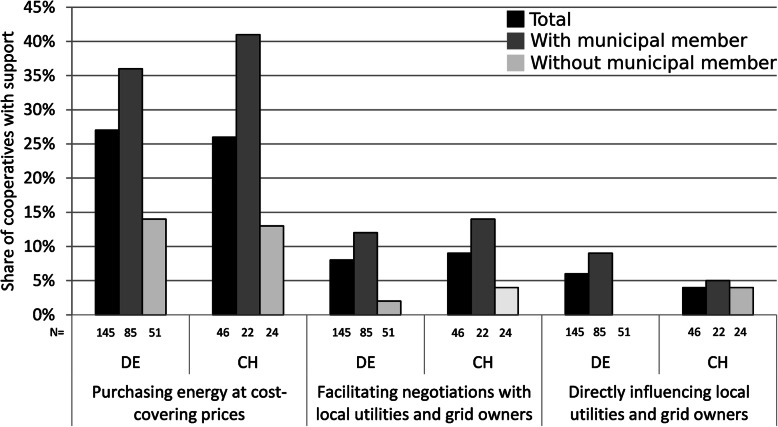
Municipal support with and without municipal membership in Germany and Switzerland: purchasing energy at cost-covering prices, facilitating negotiations, or directly influencing local utilities and grid owners. Source: Own surveys

Given these major challenges in the commercialization of the generated electricity, the energy cooperatives in both countries were in need of municipal support in this area. Municipalities in Germany occasionally supported cooperatives by purchasing their produced energy (27%; Fig. 8), and this kind of support was significantly associated with municipal membership (see Supplementary Table A.1, Additional file 1): 36% of the cooperatives with municipal membership mentioned this kind of support as opposed to only 14% of those cooperatives without municipal membership. In Switzerland, 26% of energy cooperatives mentioned municipal support in the form of purchasing their generated electricity. Whereas this total was only marginally lower than in Germany, the difference between energy cooperatives with and without municipal membership was much larger (41% vs. 13%). Other kinds of support in commercialization by municipalities, such as facilitating negotiations or directly influencing local utilities and grid owners, were only rarely mentioned in either Germany or Switzerland (Fig. 8).

#### Cooperation and expertise

We expected insufficient professional expertise to be a major limiting factor since energy cooperatives in Germany (81%) and Switzerland (87%) depended on voluntary work and 66% of the German and 72% of the Swiss cooperatives did not have any salaried positions (Table 6). However, this was not reflected in our data. Only 12% of the German and 10% of the Swiss cooperatives mentioned that their own expertise had been a limiting factor thus far. Municipal support by providing expertise in energy-related issues was rarely mentioned in either Germany (3%) or Switzerland (2%; Fig. 4).

**Table 6:** Importance of voluntary work for energy cooperatives in Germany and Switzerland. Source: Own surveys

Variables	Country					
	Germany			Switzerland		
	Total	Municipality		Total	Municipality	
		Member	No member		Member	No member
Strong dependency on voluntary work	81% (128 out of 158)	82% (72 out of 88)	79% (46 out of 58)	87% (41 out of 47)	92% (22 out of 24)	83% (19 out of 23)
No salaried positions	66% (104 out of 158)	67% (59 out of 88)	64% (37 out of 58)	72% (34 out of 47)	60% (13 out of 22)	84% (21 out of 25)

Interestingly, German cooperatives more often assessed their own expertise as a major limiting factor for the future as compared with their Swiss counterparts (28% vs. 9%; Fig. 3). One reason for this difference may be a larger share of German cooperatives assessed stringent legal requirements (e.g., technical or environmental standards in project development) as a major limiting factor in the future (cp. the “Support in planning and permit procedures” section). In addition, “old” business models (e.g., electricity generation by PV and remuneration through FITs) have no longer been feasible due to changes in the legal framework in Germany (cp. the Electricity markets and national support policies for renewable energies).^8^ Therefore, energy cooperatives in Germany have been challenged to develop new—and often technically and legally complex—business models (e.g., e-car-sharing, contracting), which could explain this result. This corroborates the results of Herbes et al. [44]: their German interview partners acknowledged the lack of know-how or competencies as a major constraint to business model innovation.

### Discussion

Our findings imply that municipal support matters for energy cooperatives in both countries. It occurs along all the development stages of renewable energy projects (project development, production, and selling, cp. Fig. 1), and it supplements national support policies for RE, especially if a municipality is a cooperative member.

#### Overall municipal support and limiting factors

In both countries, the most common form of municipal support is the provision of roof space or land, followed by support in planning and permit procedures, and the purchase of electricity at cost-covering prices (Fig. 4). This addresses some of the major limitations of energy cooperatives in Germany (lack of space/land, planning/approval procedures) and in Switzerland (selling generated electricity). These limitations are partly related to national energy policies (e.g., planning provisions, financial support).

Although these forms of municipal support were similarly common in both countries, the limiting factors for energy cooperatives differed: Whereas in Germany, limiting factors mainly related to aspects in project development (space for installations and approval procedures, cp. Fig. 1), Swiss cooperatives more often perceived the selling of electricity as a major limitation. These differences reflect the large usage of land and space for renewable energy projects and local opposition against them in Germany on the one hand and the electricity market structures and support policies for renewable energies in Switzerland on the other hand. Despite these differences, the similarity of municipal support measures suggests that these measures are rather typical and/or easy to implement and hardly depend on the degree of necessity and the limiting factors.

Somewhat unexpected is the strong engagement of German municipalities in purchasing energy at cost-covering prices, since selling the produced electricity was not often considered a limiting factor by the German cooperatives. An explanation could be that municipalities purchase at cost-covering prices to foster the development of renewable energies by local actors and to reduce (their) CO_2_ emissions in order to fulfill energy policy and climate protection goals [81]. Because of the reduced national FITs, municipal electricity purchase at cost-covering prices is likely to become economically vital to electricity-producing cooperatives, as it is already the case in Switzerland.

When it comes to financial support, we did observe a link with the needs of cooperatives. The German cooperatives were rarely limited by access to debt capital while Swiss cooperatives were often so. Accordingly, municipal financial support was rare in Germany but more common in Switzerland (Figs. 4 and 5). The latter result may be explained by the difficulties of Swiss cooperatives to raise debt capital. It has also to be stressed, however, that Swiss municipalities have a higher degree of fiscal autonomy and financial self-reliance [74] and thus are able to provide financial support even more so than German municipalities (cp. the “Municipalities in the federal systems of Germany and Switzerland” section). The easy access of German cooperatives to debt capital may be explained by the low credit risk associated with the prevalence of the German FIT (cp. the “Financial support” section).

In summary, municipalities do not complement national policies in a way that specifically addresses cooperatives’ limitations with support measures. Rather, they generically support cooperatives with the options they have, thereby filling gaps left by national policies. However, these results need to be differentiated when municipal membership in cooperatives is considered.

#### Municipal support in case of municipal membership

Two insights stand out when considering municipal membership in energy cooperatives. First, municipal support is positively associated with municipal membership in both countries: In all development stages of renewable energy projects, municipalities support energy cooperatives more frequently if they are a cooperative member. For instance, energy cooperatives with municipal membership benefit more often from the purchase of generated electricity by municipalities. Second, municipalities and their support measures complement national energy policies if municipalities are a cooperative member: Our data showed that municipalities that were cooperative members provided support exactly in the areas which the cooperatives more frequently saw as limiting to their development. More specifically, energy cooperatives in Switzerland with municipal membership more frequently received financial support whereas German cooperatives almost never received financial support, independent of municipal membership. Energy cooperatives in Germany with municipal membership were more often supported by fast processing of approval procedures. These cooperatives mentioned resistance at the local level significantly less often as a major limiting factor compared to those without municipal members. This indicates that organizational involvement of a municipality in energy cooperatives may not only lead to more support but can also help legitimize cooperative renewable energy projects and their local acceptance.

Overall, this suggests that municipalities that are cooperative members provide more targeted support than non-members and thus meet the specific cooperatives’ challenges due to national energy policy. Also, municipalities that are members are better attuned to the cooperatives’ problems or have a greater vested interest in their success. Such an interest could be the cooperatives’ engagement in municipal energy policies. Indeed, many energy cooperatives are politically active [81]. Taken together, our results support the notion that politics relating to cooperatives is a multi-level governance issue and the politics of each level impact on the system [27].

#### Limitations

Five limitations concern the content of our survey and study: Firstly, we focused only on electricity-generating energy cooperatives founded after 2006, which mostly used PV. Therefore, our results are not necessarily transferable to energy cooperatives using other technologies (e.g., wind power) or being active in other fields (e.g., heat generation) due to technological and legal differences. Above, we explained why we focused on PV after 2006. Secondly, we were unable to discern whether municipal members of cooperatives support them because they are members or whether they only become members after providing support, for instance due to acquaintance or desire for influence. Thirdly, municipalities can indirectly support energy cooperatives through municipal utilities [41, 44]. This kind of support was not included in our survey and analysis unless it was regarded as a kind of (direct) municipal support by the cooperatives. Fourthly, we did not consider state and county legislation which could provide further insights regarding support of energy cooperatives. Fifthly, we reiterate that our data and analysis reflect the situation up until 2016. In the meantime, the energy law in both countries has changed and further amendments may come up soon in this fast-evolving policy field, which we did not take into account here.

Further limitations concern methodical issues. Our examination of the limiting factors is based on the perception of the survey participants, usually a single member from the executive or supervisory board of the cooperative (cp. the “Survey design, data analysis, and quality” section). The answers are therefore subjective. For example, it may have been difficult for the survey participants to assess their own expertise and to consider it as a limiting factor (cp. the “Cooperation and expertise” section). Nevertheless, we consider the answers to be valid because there are hardly outliers and they reflect discussions and other studies. Another potential limitation is that our survey results of municipal support are based solely on information provided by the cooperatives and not by the municipalities. However, we conducted qualitative interviews with municipal actors which were not included in this paper but corroborate the information about municipal support provided by the cooperatives [81].

Finally, we assume that our insights are transferable to other countries—though to federal systems where municipalities or other local governments also have a high degree of local autonomy (cp. the “Municipalities in the federal systems of Germany and Switzerland” section [25, 70, 85]).

## Conclusion

Our analysis shows that municipal support can benefit energy cooperatives as it helps mitigating some of the major limitations of energy cooperatives in Germany and in Switzerland, some of which are directly related to the national energy policies (e.g., planning provisions, financial support). However, our data suggest that municipalities do not complement national policies in a way that specifically addresses cooperatives’ limitations with support measures. Rather, they generically support cooperatives with the options that are available to them and thus, intentionally or not, fill gaps left by national policies. However, municipalities specifically address cooperatives’ limitations with support measures if they are a member in the cooperative. This indicates that the organizational involvement of a municipality in an energy cooperative leads to a more targeted support and thus helps mitigating the specific challenges of energy cooperatives. In the future, municipal support might become even more important for energy cooperatives due to the reduced national support for renewable energies in Germany and Switzerland. On their part, by collaborating with energy cooperatives, municipalities gain an instrument to implement municipal energy policy. They may even achieve a leverage effect by supporting energy cooperatives. Hence, supporting and becoming members in energy cooperatives appear to be adequate strategies for municipalities to foster municipal energy provision and so a decentralized energy transition.

Regarding research on community energy, this paper illustrates that additional insights can be gained if support structures from more than one governmental level are analyzed. By including both the national and local levels, it was possible to elaborate the complementary function of the municipalities to national energy policy if the latter are cooperative members. Yet, further questions arise. Our analysis used quantitative data and statistical analyses that leave open questions about underlying mechanisms and motives. For instance, it remains unclear how municipal membership increases the recognition of cooperatives’ needs: Is it a matter of improved knowledge about the cooperatives’ particular situations or rather a matter of vested interests that come with financial participation? Qualitative research is required to answer such questions and to complete the picture about the relationship between cooperatives and municipalities and about underlying motivations and mechanisms. Moreover, there were significant variations within the investigated cases in both countries. It would be worthwhile to take a closer look at certain outliers, for example at cooperatives that are successful without municipal support or at municipalities that heavily support cooperatives without being members of the cooperative. Finally, our paper indicates that energy cooperatives and community energy organizations in general should not be perceived as isolated, purely civil society phenomena. Rather, there are often various ties with public actors, and such ties seem to be important to the existence and success of cooperatives.

### Supplementary information

**Additional file 1: Supplementary Table A.1.** *χ*^2^ test for associations/Fisher’s exact test. Source: Own surveys.

## Data Availability

The datasets used and/or analyzed during the current study are available from the corresponding author on reasonable request.

## Abbreviations

CH: Switzerland
CHF: Swiss franc
DE: Germany
EEG: Renewable Energy Sources Act (Erneuerbare Energien Gesetz)
FIT: Feed-in tariff
GenG: Cooperative law (Genossenschaftsgesetz)
KEV: Cost-covering remuneration for feed-in to the electricity grid (Kostendeckende Einspeisevergütung)
kWp: Kilowatt peak
MWh: Megawatt hour
PV: Photovoltaic

## Footnotes

1 With regard to the share of the specific energy sectors: electricity, heat, and mobility ([28], p., 37]).

2 If the expected frequency of one cell in *χ*^2^-test is lower than 5, we applied Fisher’s exact test [58].

3 The estimate is based on the percentage of cooperatives in our survey using PV and their mean installed PV capacity. We proportionately projected these numbers to all active energy cooperatives in Germany and Switzerland (based on DGRV [47] and the Swiss trade register), resulting in the aggregated amount of installed PV capacity by all energy cooperatives. The overall share of installed PV capacity is based on the data of the BMWi [60] and the SFOE [30].

4 Note that the revisions of the Swiss energy law of 2018 were large, though the support for small-scale producers remains limited.

5 KEV: Kostendeckende Einspeisevergütung

6 Answers with regard to future limitations are, however, very uncertain. In these questions, many cooperatives answered “don’t know” (stringent requirements: *n* = 20; duration of approval procedure: *n* = 34). These numbers are NOT represented in the percentage.

7 A possible explanation might be cooperatives’ technological focus on PV and their limited activities in the more conflictual wind energy.

8 As the EEG guarantees the FITs for 20 years, this only relates to new projects.
