# Supplementary material for: How municipalities support energy cooperatives: survey results from Germany and Switzerland
Source: Energy Sustain Soc. 2020 Mar 18;10(1):18. doi: 10.1186/s13705-020-00248-3 (PMC7081670; doi:10.1186/s13705-020-00248-3)
Supplement: Supplementary file 1 — Additional file 1: Supplementary Table A.1. χ2 test for associations/Fisher’s exact test. Source: Own surveys. [file 13705_2020_248_MOESM1_ESM.pdf]

Supplementary Table A.1: Chi2-test for associations / Fisher's exact test. Source: Own surveys.

Chi²-test for associations / Fisher's exact test												
	**Comparison between (Independent variable)**											
	Country				Municipal membership in DE				Municipal membership in CH			
**Comparison of (Dependent variable)**	$\chi 2$	N	p (two-sided)[a)c)]	Cramer's V[d)]	$\chi 2$	N	p (two-sided)[a)c)]	Cramer's Vd[)]	$\chi 2$	N	p (two-sided)[a)c)]	Cramer's Vd[)]
**Support by municipality; df=1 for all tests**												
Granting of a loan	b)	201	.011*	.202	b)	144	.153	.137	b)	46	.090	.275
Guarantee for loans from third parties	b)	201	.080	.141	b)	144	1.000	.068	b)	46	.101	.276
Other kind of financial support / Financial support in other forms (e. g. municipal energy fund)	b)	201	.002**	.262	n/a	144	n/a	n/a	b)	46	1.000	.013
Any kind of financial support (Pooling)	b)	201	.000**	.345	b)	144	.153	.137	4.804	46	.028*	.323
Provision of (roof) space for PV	.030	201	.863	.012	3.284	144	.070	.151	1.344	46	.246	.171
Support through administrative procedures / Fast processing of licensing procedures	1.488	201	.222	.086	5.760	144	.016*	.200	1.315	46	.251	.251
Purchasing energy at cost-covering prices	.016	201	.899	.009	9.667	144	.002**	.259	4.804	46	.028*	.323
Facilitating negotiations with local utilities and grid owners	b)	201	.751	.026	b)	144	.050*	.179	b)	46	.336	.168
Directly influencing local utilities and grid owners	b)	201	1.000	.016	b)	144	.022*	.196	b)	46	1.000	.009
Expertise in energy issues	b)	201	1.000	.016	b)	144	.153	.137	b)	46	.478	.156
**Limiting factors (so far) (no=0; 1=yes); df=1 for all tests**												
Access to debt capital	b)	153	.004**	.252	b)	114	.481	.091	b)	33	1.000	.012
Acquisition of roof space for PV	17.617	170	.000**	.322	.355	125	.551	.053	b)	37	.251	.236
Finding of locations for other RE	9.095	139	.003**	.256	.895	104	.344	.093	b)	28	.410	.204
Stringent legal requirements / Stringent requirements on projects due to legal regulations (technical standards, environmental standards, etc.)	15.231	162	.000**	.307	1.838	119	.175	.124	b)	37	1.000	.017
Long duration of proceedings for the construction of new facilities due to objections	6.089	145	.014*	.205	4.743	103	.029*	.215	b)	37	1.000	.028
(Political) resistance at the local level	6.413	157	.011*	.202	6.503	114	.011*	.239	b)	36	1.000	.084
Sale of produced energy at cost-covering prices / Lack of sales opportunities for energy produced at cost-covering prices	7.900	160	.005**	.222	.190	114	.663	.041	0.921	40	.337	.152
(Own) expert knowledge e (business, technical, legal)	b)	162	1.000	.026	.334	116	.563	.054	b)	39	1.000	.026

**Limiting factors (future) (no=0; 1=yes); df=1 for all tests**												
Access to debt capital	b)	119	.000**	.420	b)	85	.148	.171	.007	28	.934	.016
Acquisition of roof space for PV	10.466	139	.001**	.274	4.676	101	.031*	.215	4.097	32	.043*	.358
Finding of locations for other RE	6.281	118	.012*	.231	.299	87	.585	.059	3.222	25	.073	.359
Stringent legal requirements / Stringent requirements on projects due to legal regulations (technical standards, environmental standards, etc.)	19.277	132	.000**	.382	.196	95	.658	.045	.622	32	.430	.139
Long duration of proceedings for the construction of new facilities due to objections	19.793	105	.000**	.434	2.333	70	.127	.183	b)	30	1.000	.026
(Political) resistance at the local level	5.667	108	.017*	.229	5.295	74	.021*	.267	b)	28	.600	.175
Sale of produced energy at cost-covering prices / Lack of sales opportunities for energy produced at cost-covering prices	7.394	121	.007**	.247	.002	83	.961	.005	b)	32	.672	.101
(Own) expert knowledge e (business, technical, legal)	5.055	128	.025*	.199	.001	86	.971	.004	b)	34	.571	.014
**Member groups (no member=0; member=1); df=1 for all tests**												
Membership of municipality	1.572	198	.210	.089								
Membership of cooperative bank	31.610	198	.000**	.400	34.658	148	.000**	.484	b)	50	.110	.295
Membership of other bank	13.748	198	.000**	.263	13.238	148	.000**	.299	b)	50	1.000	.143
**Financial characteristics (no=0; yes=1); df=1 for all tests**												
Use of debt capital	10.886	202	.001**	.232	.223	144	.637	.039	1.466	46	.226	.179
Difficulties to raise debt capital (very or rather difficult)	4.508	153	.034*	.172	.001	118	.970	.003	b)	25	.434	.175
Loans from cooperative banks	23.805	202	.000**	.343	1.259	144	.308	.094	b)	46	.019*	.365
**Voluntary work and salaried positions (no=0; yes=1); df=1 for all tests**												
Strong dependency on voluntary work	.968	205	.325	.069	0.142	146	.706	.031	b)	47	.416	.136
No salaried positions	.699	205	.403	.058	0.164	146	.685	.034	3.629	47	.057	.278

a) For the evaluation of significance we applied Chi^2-test (Pearson)
b) Fisher's exact test if the expected frequency of one cell in Chi^2-test is lower than 5
c) *: p<0.05; **: p<0.01
d) Cramer’s V measures the strength of an association: values .10, .30 and .50 correspond to small, medium and large associations respectively
